# Risk factors for electrical storms following percutaneous coronary intervention in patients with acute myocardial infarction: A meta-analysis

**DOI:** 10.17305/bb.2024.10274

**Published:** 2024-10-01

**Authors:** Xiao Xiong, Qiang Ye, Yongquan Peng

**Affiliations:** 1 Department of Cardiology, The Affiliated Hospital of Southwest Medical University, Luzhou, China

**Keywords:** Electrical storm (ES), percutaneous coronary intervention (PCI), acute myocardial infarction (AMI), risk factors, β-blockers

## Abstract

Electrical storms (ESs) following percutaneous coronary intervention (PCI) in acute myocardial infarction (AMI) patients pose a significant challenge, affecting prognostic outcomes and increasing mortality. This meta-analysis synthesized data from 11 studies involving 9666 AMI patients to identify risk factors associated with ES following PCI. Our findings revealed an average ES incidence of 7.70%, with identified risk factors, including low thrombolysis in myocardial infarction (TIMI) flow grades (0–1), elevated cardiac troponin I (cTnI) levels, persistent hypotension, reperfusion arrhythmias, the right coronary artery being the infarct-related artery (IRA), increased diameter of the IRA, renal dysfunction, elevated creatine kinase-MB (CK-MB), and bradycardia. Notably, the use of β-blockers was found to significantly reduce the risk of ES. The study underscores the importance of early identification and management of these risk factors in AMI patients undergoing PCI to prevent the occurrence of ES, highlighting the protective role of β-blockers. This research provides a foundation for future strategies aimed at reducing the incidence and improving the prognosis of ES in this patient population.

## Introduction

Ventricular tachycardia (VT) and electrical storms (ESs), defined as the occurrence of two or more episodes of VT or ventricular fibrillation (VF) within 24 h, are clinical syndromes resulting from unstable cardiac electrophysiology, leading to malignant ventricular arrhythmias [1, 2]. These conditions are frequently observed in patients with acute myocardial infarction (AMI), ischemic heart disease with a history of myocardial infarction, cardiomyopathies, valvular heart disease, catecholaminergic polymorphic VT, and heart failure [3–8].

Myocardial ischemia, a significant trigger for ES, causes changes in cardiac ion channels and activates the sympathetic nervous system, leading to an influx of catecholamines, β-receptor activation, and increased myocardial repolarization dispersion, thereby inducing VT and VF [9]. AMI also enhances the automaticity of Purkinje fibers, elevating the risk of malignant ventricular arrhythmias. Ischemia-induced cardiac structural changes, including ventricular chamber enlargement, deformation, and compensatory hypertrophy of non-infarcted areas, contribute to cardiac remodeling [10].

Meanwhile, cardiac remodeling impairs cardiac contractility, exacerbates heart failure, and further deteriorates left ventricular function through repeated VT and VF episodes, thereby inducing malignant arrhythmias [11]. Clinically, ES complicates 10%–20% of AMI cases [12]. The MADIT II study confirmed that AMI patients have a 3.1-fold higher risk of experiencing ES compared to healthy individuals, with some presenting ES as an initial symptom [13].

With the aging population in China, the incidence of AMI is rising annually, exceeding 500,000 new cases each year and showing a trend toward younger age groups, posing a significant threat to public health [14, 15]. Percutaneous coronary intervention (PCI) is the recommended treatment for AMI, effectively restoring blood flow to the infarcted myocardium [16]. However, persistent VT following PCI treatment has been reported, making AMI patients a high-risk group for ES, even after PCI. ES significantly increases the risk of sudden death, with mortality rates 7.4 times higher than those without ES [17]. Increasing evidence indicates that ES is a predictor of poor outcomes in AMI patients [18]. The clinical management of ES is challenging, with high mortality rates persisting despite long-term prevention through implantable cardioverter-defibrillator insertion and pharmacological treatments, with a 2-year mortality rate exceeding 20% [19].

Therefore, investigation into the risk factors for ES following PCI in AMI patients and elucidating the mechanisms of ES onset can facilitate early risk prediction and the development of effective treatment strategies. Early intervention and precision medicine could reduce the incidence and mortality of ES, improving patients’ quality of life and alleviating societal burdens. Despite increasing research on risk factors in ES following PCI, studies remain limited, and results are inconsistent, with no consensus on some findings. Many studies lack comprehensive risk factor analysis and suffer from small sample sizes and unstable results. This study aims to evaluate the risk factors for ES following PCI in AMI patients through meta-analysis, providing strong evidence to reduce the occurrence of ES following PCI and improve patient outcomes.

## Materials and methods

### Database retrieval

A computer-based web search was conducted to retrieve literature published in both Chinese and English databases. The Chinese databases included the China National Knowledge Infrastructure (CNKI), Wan Fang Database, the Chinese Scientific Journals Full-Text Database (VIP), and the China Biological Medicine Database (CBM). English databases comprised PubMed, Web of Science, Embase, and the Cochrane Library.

The search strategy employed a combination of subject headings and free-text terms. Chinese search terms included: AMI, PCI, sympathetic storm, VT storm, ventricular arrhythmia storms, ES, persistent VT, persistent VF, risk factors, logistic regression analysis, and Cox regression analysis. English search terms representing these concepts were used.

### Inclusion and exclusion criteria

Inclusion criteria were: (1) publications from international databases consisting of cohort studies, case-control studies, and cross-sectional studies; (2) diagnosis of AMI adhering to the diagnostic criteria outlined in the 2017 guidelines by the American Heart Association/American College of Cardiology/European Society of Cardiology, including elevation and subsequent decline of myocardial necrosis biomarkers (cardiac troponin I [cTnI] or creatine kinase [CK]-MB) alongside at least one of the following: symptoms of myocardial ischemia, pathological Q waves, or ST-segment elevation or depression; (3) publications in either Chinese or English; (4) completeness of patient clinical data, including specific case numbers and details, with complete and reliable follow-up data if available; (5) similarity in study methods and objectives, specifically aiming to explore risk factors for ES following PCI in AMI patients; (6) completeness of study outcomes and statistical results with corresponding interpretations; and (7) a sample size of no less than 40 cases.

Exclusion criteria included: (1) duplicate publications or those irrelevant to the topic; (2) studies with insufficient sample sizes (less than 40 cases); (3) studies with unclear research subjects or non-compliance with relevant diagnostic standards; (4) reports with minimal information, unclear data description, unclear data sources, inability to extract effective outcomes, or inability to access the full text; (5) animal experiments or in vitro studies; and (6) meta-analyses, case reports, expert commentaries, reviews, conference papers, guidelines, and consensus statements.

### Evaluation of literature quality

Following the inclusion and exclusion criteria, all retrieved literature from the databases was screened collaboratively by two researchers. The quality of the literature was assessed using the Newcastle–Ottawa Scale (NOS), with a full score of 9 points. Publications scoring ≥6 were considered qualified, while those scoring <6 were excluded. For publications with discrepant evaluations, a third experienced researcher (a graduate supervisor) was consulted for joint analysis and guidance, ultimately determining inclusion.

### Data extraction

The Endnote X9 software was utilized to manage and extract information from the included literature, capturing author information (first author), publication year, country, sample size, type of study, and quality assessment outcomes, along with identifying potential risk factors.

### Ethical statement

This article does not contain any research with human participants or animals performed by any of the authors.

### Statistical analysis

The “Meta” package in R language was used for meta-analysis and statistical testing of the selected literature. Continuous variables were represented by the standardized mean difference (SMD), and dichotomous variables by the relative risk (RR). Heterogeneity was assessed using the Q test and *I*^2^ statistics, with *I*^2^ ≥ 50% indicating significant heterogeneity and *I*^2^ < 50% indicating no significant heterogeneity. A random-effects model was applied for studies with significant heterogeneity, while a fixed-effects model was used for those without. Further sensitivity analyses were conducted. A random-effects model was applied for studies where heterogeneity was within acceptable limits, but the source of heterogeneity could not be identified. Descriptive analysis was performed for studies with particularly strong heterogeneity.

## Results

### Basic characteristics and quality assessment of included studies

A total of 1342 relevant articles were selected in this screening process. Management and screening of these articles were facilitated using Endnote, which resulted in the exclusion of 116 duplicates. Furthermore, 484 articles were excluded, including conference papers, reviews, case reports, guidelines, and expert commentaries. Upon a more detailed review of titles and abstracts in both English and Chinese, 654 articles that did not align with the objectives of this study were also excluded, leading to the inclusion of 88 articles. A full-text examination led to the further exclusion of 58 articles due to incomplete information or poor quality, two articles for duplicity in language, and 17 articles lacking comprehensive statistical results, including OR and 95% confidence intervals. Ultimately, 11 articles were included for meta-analysis [20–30].

**Figure 1.**
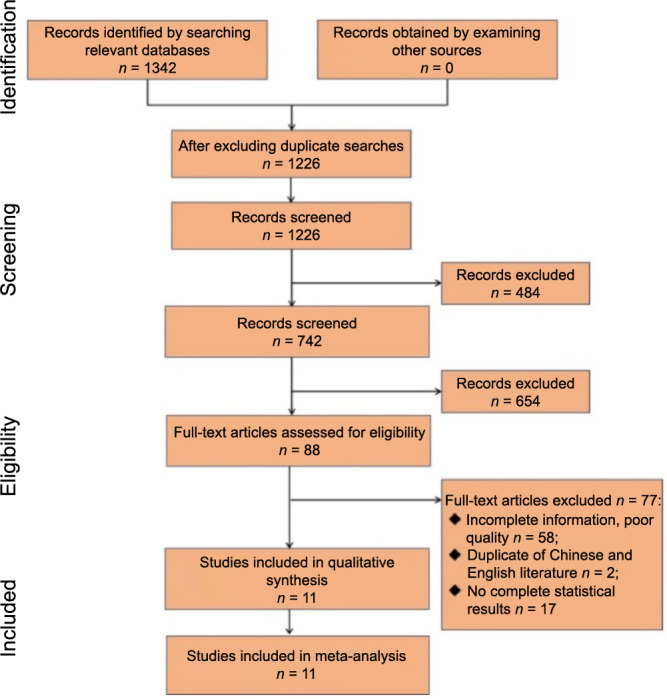
Flowchart of literature selection process.

The scarcity of English literature on AMI complicated by ES necessitated a focus on studies primarily from Chinese databases, with the majority of included studies being in Chinese and only one in English. The selection process is depicted in Figure 1.

From the selected 11 articles, 17 risk factors were identified, including cTnI, TIMI flow grades, persistent hypotension, ECG J-waves, creatine kinase-MB (CK-MB), infarct-related artery (IRA), IRA diameter, reperfusion arrhythmias, BNP, renal insufficiency, newly developed atrioventricular block, ST-segment depression less than 70%, baseline heart rate greater than 70/min, post-PCI TIMI grading, use of blockers within 24 h, left ventricular ejection fraction ≤35%, and a TIMI risk score of 8–14. These risk factors were identified from perioperative events reported in all included articles.

The incidence of ES across the included studies ranged from 5.70% to 21.46%, with a total sample size of 9666 and 744 instances of ES, resulting in an average incidence rate of 7.70%. The baseline characteristics of the included studies are detailed in Table 1, and clinical baseline characteristics are presented in Table 2.

**Table 1:** Basic characteristics of the included studies

**First author**	**Year**	**N**	**ES incidence**	**Diseases**	**Risk factors**
Wang ZM [16]	2018	142	28 (19.72%)	Acute myocardial infarction	ECG J wave, TNI values, TIMI flow grade, and persistent hypotension
Yao J [17]	2016	2343	183 (7.81%)	ST-segment elevation myocardial infarction	Left ventricular ejection fraction 35%, renal function, use of β -blockers, TIMI risk score 8 to 14
Jiao FX [18]	2015	168	23 (13.69%)	Acute ST-segment elevation myocardial infarction	CK-MB value, TNI values, IRA, TIMI flow grade, and persistent intraoperative hypotension
He SH [19]	2014	280	19 (6.79%)	Acute myocardial infarction	TNI values, TIMI grade, and persistent hypotension
Sun QB [20]	2014	253	52 (20.55%)	Acute ST-segment elevation myocardial infarction	Reperfusion with arrhythmia, BNP, and renal insufficiency
Zhang J [21]	2013	120	11 (9.17%)	ST-segment elevation type of myocardial infarction	CK-MB value, TNI value, IRA was right coronary artery, TIMI grade, bradycardia (less than or equal to 45 beats/min), and persistent hypotension
Xu JR [22]	2012	41	7 (17.07%)	Acute myocardial infarction	IRA diameter, TIMI flow grade, and reperfusion arrhythmia after opening of the IRA
Li XT [23]	2011	114	9 (7.9%)	ST-segment elevation myocardial infarction	CK-MB values, TNI values, and IRA was the right coronary artery, TIMI flow grade, bradycardia, and persistent hypotension
Zhou T [24]	2010	228	39 (17.11%)	Acute myocardial infarction	IRA diameter, flow TIMI grade after IRA opening, and reperfusion arrhythmia
Liu JN [25]	2009	205	44 (21.46%)	Acute myocardial infarction	Reperfusion arrhythmia and newly emerging AV block
Rajendra H [26]	2009	5772	329 (5.7%)	ST-segment elevation myocardial infarction	Persistent hypotension, less than ST segment, 70% decrease, baseline heart rate greater than 70/min, total baseline ST deviation, grade 3 TIMI flow after PCI, grade 0 TIMI flow before PCI within 24 hours

N: Case number; ES: Electrical storm; ECG: Electrocardiogram; PCI: Percutaneous coronary intervention; TIMI: Thrombolysis in myocardial infarction; TNI: Troponin I; AV block: Atrioventricular block; CK-MB: Creatine kinase isoenzyme; IRA: Infarct-related artery; BNP: B-type natriuretic peptide.

**Table 2:** Basic characteristics of patients in the included studies

**First author**	**Year**	**ES**						**Non-ES**					
		**Ages (Y)**	**Male**	**HBP**	**Diabetes**	**Hyperlipidemia**	**Smoking**	**Ages (Y)**	**Male**	**HBP**	**Diabetes**	**Hyperlipidemia**	**Smoking**
Wang ZM [16]	2018	72.58 ± 7.32	18 (64.3)	18 (64.3)	16 (57.1)	15 (53.6)	15 (53.6)	70.84 ± 5.26	75 (65.8)	72 (63.2)	68 (59.6)	54 (47.4)	60 (52.6)
Yao J [17]	2016	59.2 ± 11.4	158 (86.3)	97 (53.0)	57 (31.1)	NA	121 (66.1)	57.8 ± 12.2	633 (75.6)	1162 (53.8)	580 (26.9)	NA	1330 (61.6)
Jiao FX [18]	2015	56.2 ± 10.5	18 (78.3)	NA	NA	NA	NA	57.4 ± 9.7	119 (82.1)	NA	NA	NA	NA
He SH [19]	2014	62.4 ± 5.1	12 (63.2)	18 (94.5)	7 (36.8)	16 (84.2)	10 (52.6)	63.6 ± 57.2	172 (65.9)	166 (63.6)	92 (35.2)	156 (59.8)	78 (29.9)
Sun QB [20]	2014	63.44 ± 10.71	40 (76.9)	NA	NA	NA	NA	59.82 ± 10.83	155 (77.1)	NA	NA	NA	NA
Zhang J [21]	2013	**Undefined**											
Xu JR [22]	2012	70.3 ± 9.8	NA	NA	NA	NA	NA	51.5 ± 12.3	NA	NA	NA	NA	NA
Li XT [23]	2011	56.1 ± 11.5	8 (88.9)	4 (44.4)	2 (22.2)	5 (55.6)	4 (44.4)	58.4 ± 10.9	83 (79.0)	50 (47.6)	22 (21.0)	53 (50.5)	44 (3.8)
Zhou T [24]	2010	69.7 ± 10.3	32 (82.1)	NA	NA	NA	NA	50.9 ± 12.8	135 (71.4)	NA	NA	NA	NA
Liu JN [25]	2009	31 (>60)	40 (90.1)	5 (11.3)	17 (38.6)	NA	NA	86 (>60)	131 (81.4)	21 (13.0)	66 (41.0)	NA	NA
Rajendra H [26]	2009	64 (53-72)	239 (75.4)	162 (49.2)	61 (18.5)	131 (52.8)	134 (41.0)	61 (52-71)	4278 (77.0)	2677 (49.4)	852 (15.7)	2049 (49.5)	2344 (43.4)

Data are given as mean ± SD or count (%). ES: Electric storm; HBP: High blood pressure; NA: Not available.

The quality of the included 11 articles was evaluated using NOS (Table 3). According to the Cochrane Handbook, these articles scored between 6 and 8 points, qualifying as acceptable quality.

**Table 3:** The scoring of included studies was conducted using The Newcastle-Ottawa Scale

**First author**	**Year**	**Selection of study population**				**Comparability between groups**	**Measurement of exposure factors**			**Scores**
		①	②	③	④		⑤	⑥	⑦	
Wang ZM [16]	2018	1	1	1	1	1	1	1	1	8
Yao J [17]	2016	0	1	1	1	1	1	1	1	7
Jiao FX [18]	2015	1	1	1	1	1	1	1	0	7
He SH [19]	2014	1	1	1	1	1	1	1	1	8
Sun QB [20]	2014	1	1	1	1	1	1	1	0	7
Zhang J [21]	2013	1	0	1	1	1	1	1	0	6
Xu JR [22]	2012	1	0	1	1	1	1	1	0	6
Li XT [23]	2011	1	1	1	1	1	1	1	1	8
Zhou T [24]	2010	1	1	1	1	1	1	1	1	8
Liu JN [25]	2009	1	1	1	1	1	1	1	1	8
Rajendra H [26]	2009	1	1	1	1	1	1	1	1	8

Note: ① Appropriateness of cases; ② Representativeness of cases; ③ Selection of controls; ④ Determination of controls; ⑤ Determination of factors; ⑥ Same method; ⑦ Non-response rate.

### Correlation between TIMI flow grades 0–1 and ES following PCI in AMI

**Figure 2.**
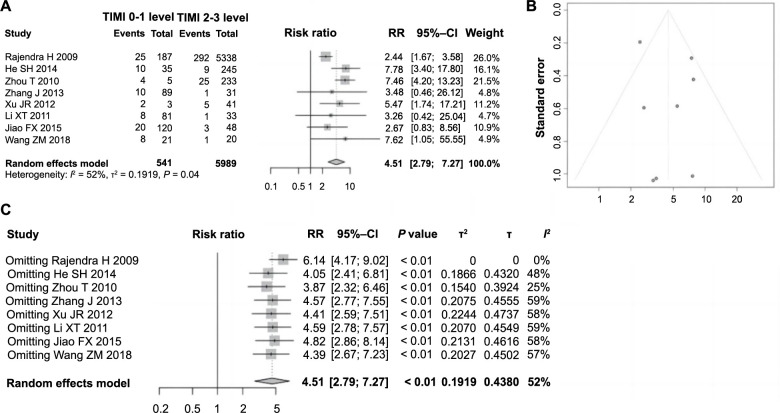
**Forest plot of meta-analysis on the association between TIMI blood flow grade and ES following PCI in AMI.** (A) Forest plot of TIMI blood flow grading and ES following PCI in AMI; (B) Evaluating publication bias using a funnel plot; (C) Sensitivity analysis. TIMI: Thrombolysis in myocardial infarction; ES: Electric storm; PCI: Percutaneous coronary intervention; AMI: Acute myocardial infarction.

**Figure 3.**
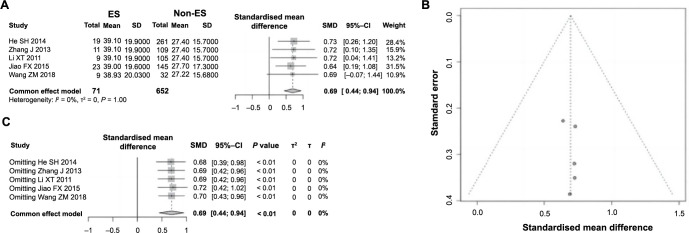
**Forest plot of meta-analysis on the association between cTnI and ES following PCI in AMI.** (A) Forest plot depicting the association between cTnI and ES following PCI in AMI; (B) Funnel plot assessing publication bias; (C) Sensitivity analysis. cTnI: Cardiac troponin I; ES: Electric storm; PCI: Percutaneous coronary intervention; AMI: Acute myocardial infarction; RR: Relative risk; CI: Confidence interval.

Among the included articles, eight examined the impact of TIMI flow grades on the occurrence of ES following PCI for AMI, involving 6530 patients. Of these, 541 had a TIMI flow grade of 0–1, while 5989 had a TIMI flow grade of 2–3. Heterogeneity testing revealed significant variability across studies (*P ═* 0.04, *I*^2^ *=* 52%), necessitating the use of a random-effects model for analysis. The meta-analysis indicated a significant association, with a combined RR of 4.51 (95% CI: 2.79–7.27, *P* < 0.01), suggesting a higher incidence of ES following PCI in patients with TIMI flow grades 0–1 compared to those with grades 2–3 (Figure 2A). This finding indicates a significant correlation between TIMI flow grades 0–1 and the occurrence of ES, identifying it as a risk factor. The symmetry of the funnel plot suggests the absence of notable publication bias, affirming the reliability of the meta-analysis results (Figure 2B). Further sensitivity analysis did not reveal significant changes in heterogeneity among the studies, reinforcing the robustness and reliability of the meta-analysis results (Figure 2C) TnI is a risk factor for ES following PCI in AMI. Among the included 11 studies, a total of five studies analyzed the impact of cTnI on ES following PCI in AMI. These studies involved a total of 723 patients, among whom 71 experienced ES, while 652 did not. Heterogeneity testing revealed no significant variability across the studies (*P ═* 1.00, *I*^2^ ═ 0% <50%), prompting the application of a fixed-effect model for pooled analysis. As depicted in Figure 3A, patients with ES exhibited a significant increase in cTnI compared to those without ES (SMD ═ 0.69, 95% CI: 0.44–0.94; *P <* 0.01). This association underscores the significant relationship between cTnI levels and the occurrence of ES following PCI in AMI, establishing it as a risk factor. The symmetry of the funnel plot suggested the absence of substantial publication bias (Figure 3B). Sensitivity analyses further indicated no significant change in heterogeneity among the studies (Figure 3C), confirming the robustness and reliability of this conclusion.

### Correlation between persistent malignant hypotension and ES following PCI in AMI

**Figure 4.**
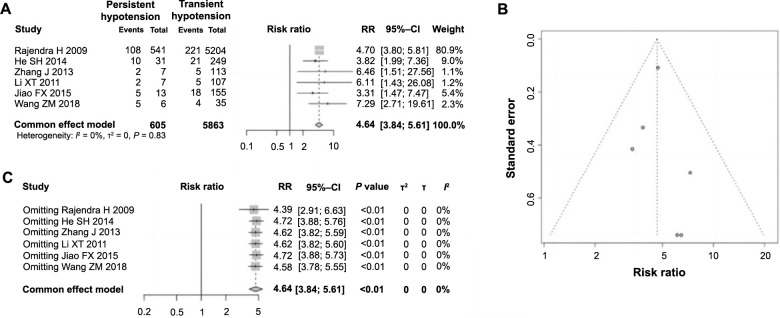
**Forest plot of meta-analysis on the association between persistent hypotension and ES following PCI in AMI.** (A) Forest plot of the association between persistent hypotension and the occurrence of ES following PCI in AMI; (B) Funnel plot for evaluating publication bias; (C) Sensitivity analysis. ES: Electric storm; PCI: Percutaneous coronary intervention; AMI: Acute myocardial infarction; RR: Relative risk; CI: Confidence interval.

Of the 11 studies included, six analyzed the impact of persistent hypotension on ES following PCI in AMI. A total of 6468 patients were included, with 605 experiencing persistent hypotension and 5864 not experiencing it. Heterogeneity testing showed no significant disparities among the studies (*P ═* 0.83, *I^2^* ═ 0% < 50%), leading to the use of a fixed-effect model for analysis. The meta-analysis results demonstrated a significant increase in the incidence of ES among patients with persistent hypotension compared to those without (RR ═ 4.64, 95% CI: 3.84–5.61, *P <*0.01), indicating a clear correlation between persistent malignant hypotension and the occurrence of ES, identifying it as a risk factor (Figure 4A). The funnel plot was essentially symmetrical, indicating minimal publication bias (Figure 4B). Further sensitivity analysis revealed no notable changes in heterogeneity across the studies (Figure 4C), validating the stability and dependability of the meta-analysis outcomes.

### Correlation between reperfusion arrhythmia and ES following PCI in AMI

**Figure 5.**
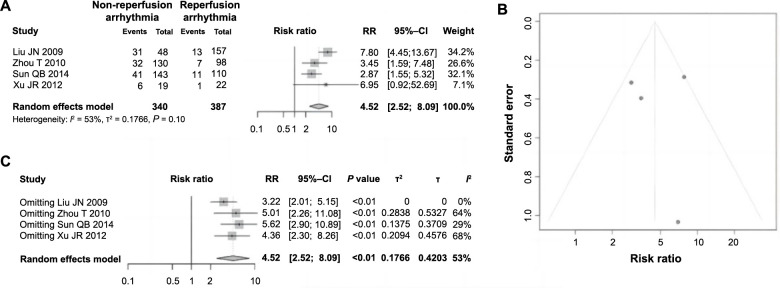
**Forest plot of meta-analysis on the association between reperfusion arrhythmia and ES following PCI in AMI.** (A) Forest plot of the association between reperfusion arrhythmia and ES following PCI in AMI; (B) Funnel plot assessing publication bias; (C) Sensitivity analysis. ES: Electric storm; PCI: Percutaneous coronary intervention; AMI: Acute myocardial infarction; RR: Relative risk; CI: Confidence interval.

Among the 11 included articles, four studies analyzed the impact of reperfusion arrhythmia on ES following PCI in AMI. These studies, comprising 727 patients, reported that 340 patients experienced reperfusion arrhythmias, while 387 did not. Heterogeneity testing indicated statistical variability among the studies (*P ═* 0.1, *I^2^ ═* 53% > 50%), leading to the employment of a random-effects model for the analysis. The meta-analysis revealed a pooled RR of 4.52 (95% CI ═ 2.52–8.09, *P* < 0.01), demonstrating statistically significant differences (Figure 5A). This finding suggests a clear association between reperfusion arrhythmias and the incidence of ES, identifying it as a risk factor. Additionally, the symmetry of the funnel plot indicated an absence of significant publication bias (Figure 5B). Subsequent sensitivity analysis showed no significant changes in heterogeneity among the studies (Figure 5C), further affirming the robustness and reliability of the meta-analysis outcomes.

### Correlation between occlusion of the right coronary artery (RCA) and cardiac arrest

**Figure 6.**
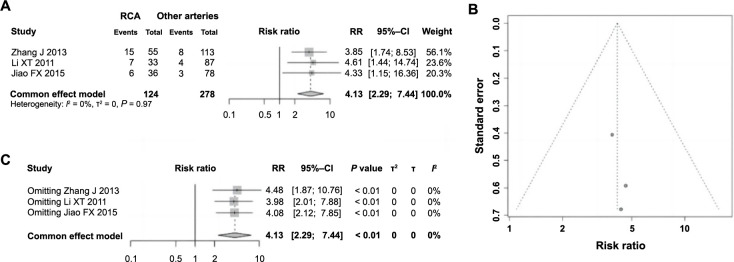
**Forest plot of meta-analysis on the association between IRA as RCA and ES following PCI in AMI.** (A) Forest plot of the IRA as the RCA and the occurrence of ES following PCI in AMI; (B) Funnel plot for assessing publication bias; (C) Sensitivity analysis. IRA: Infarct-related artery; RCA: Right coronary artery; ES: Electric storm; PCI: Percutaneous coronary intervention; AMI: Acute myocardial infarction; RR: Relative risk; CI: Confidence interval.

Among the 11 included studies, three analyzed the impact of infarct-related arteries on ES following PCI in AMI, totaling 402 patients. Of these, 124 patients had the RCA as the IRA, while 278 had other arteries implicated. Heterogeneity testing yielded no significant disparities (*P ═* 0.97, *I^2^* ═ 0% < 50%); thus, a fixed-effect model was applied for the meta-analysis. The results, as shown in Figure 6A, indicate that patients with the RCA as the IRA exhibited a significantly higher incidence of ES following PCI for AMI compared to those with other arteries involved (RR ═ 4.13, 95% CI: 2.29–7.44, *P <*0.01). This finding suggests a significant correlation between having the RCA as the IRA and the development of ES, classifying it as a risk factor. The evaluation of publication bias via funnel plot symmetry showed no significant bias (Figure 6B). Further, sensitivity analyses indicated stable heterogeneity across studies (Figure 6C), confirming the high robustness and reliability of these findings.

### Diameter of the IRA is a risk factor for ES following PCI in AMI

**Figure 7.**
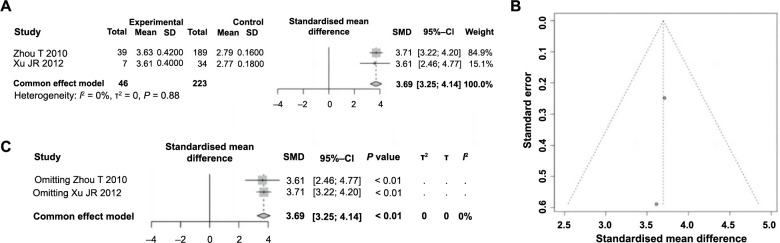
**Forest plot of meta-analysis on the association between IRA diameter and ES following PCI in AMI.** (A) Forest plot of the IRA diameter and ES following PCI in AMI; (B) Funnel plot assessing publication bias; (C) Sensitivity analysis. IRA: Infarct-related artery; ES: Electric storm; PCI: Percutaneous coronary intervention; AMI: Acute myocardial infarction; RR: Relative risk; CI: Confidence interval; SD: Standard deviation; SMD: Standardised mean difference.

Among the 11 articles included, two explored the impact of the diameter of IRA on ES following PCI in AMI. A total of 269 patients were included, with 46 experiencing ESs and 223 not experiencing them. Heterogeneity testing revealed no significant differences across the studies (*P ═* 0.88, *I^2^ ═* 0% < 50%), leading to the application of a fixed-effect model for analysis. The meta-analysis indicated a significant increase in the diameter of the IRA in patients with ES compared to those without (SMD ═ 3.69, 95% CI: 3.25–4.14; *P* < 0.01), identifying this factor as a risk factor for the development of ES following PCI in AMI (Figure 7A). A funnel plot was constructed to assess publication bias, showing no significant bias (Figure 7B). Further sensitivity analysis confirmed no significant change in heterogeneity among the studies (Figure 7C), affirming the robustness and reliability of the meta-analysis findings regarding the diameter of the IRA.

### Renal dysfunction is significantly correlated with ES following PCI in AMI

**Figure 8.**
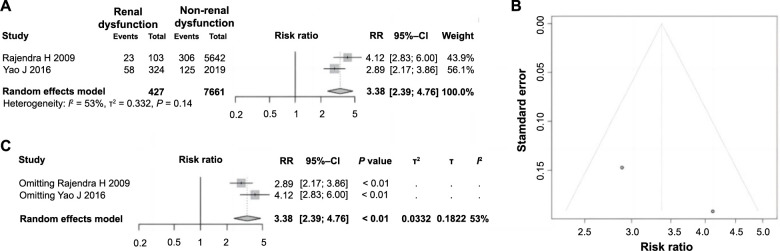
**Forest plot of meta-analysis on the association between renal insufficiency and ES following PCI in AMI.** (A) Forest plot of renal dysfunction and ES following PCI in AMI; (B) Funnel plot for evaluating publication bias; (C) Sensitivity analysis. ES: Electric storm; PCI: Percutaneous coronary intervention; AMI: Acute myocardial infarction; RR: Relative risk; CI: Confidence interval.

Among the 11 included studies, two analyzed the impact of renal dysfunction on ES following PCI in AMI. A total of 8088 patients were included in the analysis, with 427 patients having renal dysfunction and 7661 patients without it. Heterogeneity tests indicated statistical variability among the studies (*P ═* 0.14, *I^2^* ═ 53%), necessitating the use of a random-effects model for analysis. The meta-analysis revealed that patients with renal dysfunction had a significantly higher incidence of ES following PCI in AMI compared to those without renal dysfunction (RR ═ 3.38, 95% CI: 2.39–4.76, *P* < 0.01), suggesting a clear correlation between renal dysfunction and the occurrence of ES, thereby classifying it as a risk factor (Figure 8A). A funnel plot was drawn to evaluate publication bias, which indicated no apparent bias (Figure 8B). Sensitivity analysis showed that heterogeneity among the studies remained unchanged (Figure 8C), validating the reliability and stability of the meta-analysis results concerning renal dysfunction.

### Correlation between β-blockers and ES following PCI in AMI

**Figure 9.**
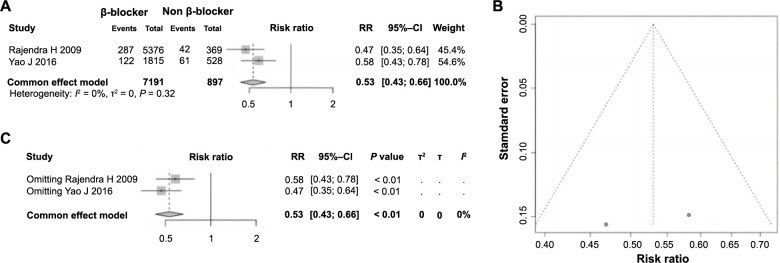
**Forest plot of meta-analysis on the association between the use of β-blockers and ES following PCI in AMI.** (A) The forest plot of renal insufficiency and ES following PCI in AMI; (B) Publication bias evaluated using a funnel plot; (C) Sensitivity analysis. ES: Electric storm; PCI: Percutaneous coronary intervention; AMI: Acute myocardial infarction; RR: Relative risk; CI: Confidence interval.

Among the 11 studies included, two analyzed the impact of β-blocker use on ES following PCI in AMI, comprising a total of 8088 patients. Among these, 7191 patients were administered β-blockers, while 897 were not. Heterogeneity testing indicated no significant disparities across the studies (*P ═* 0.32, *I^2^* ═ 0% < 50%), leading to the employment of a fixed-effect model for pooled analysis. Patients who were administered β-blockers demonstrated a significantly reduced incidence of ES following PCI in AMI compared to those who were not (RR ═ 0.53, 95% CI: 0.43–0.66, *P <*0.01), indicating a protective correlation between β-blocker usage and the occurrence of ES (Figure 9A). The funnel plot was essentially symmetrical, suggesting an absence of significant publication bias (Figure 9B). Sensitivity analysis confirmed the homogeneity among the studies remained consistent (Figure 9C), further substantiating the robustness and reliability of the meta-analysis findings.

**Figure 10.**
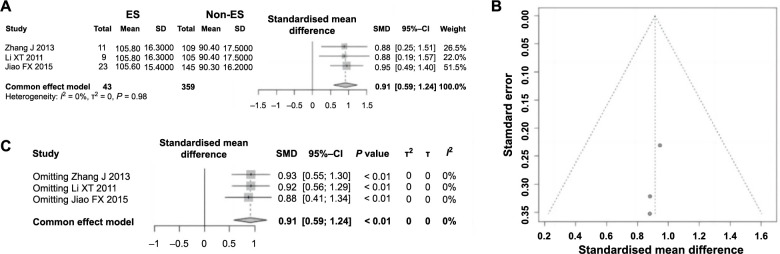
**Forest plot of meta-analysis on the association between the use of β-blockers and ES following PCI in AMI.** (A) Forest plot of CK-MB and ES following PCI in AMI; (B) Funnel plot evaluating publication bias; (C) Sensitivity analysis. ES: Electric storm; PCI: Percutaneous coronary intervention; AMI: Acute myocardial infarction; CK-MB: Creatine kinase MB; RR: Relative risk; CI: Confidence interval; SD: Standard deviation; SMD: Standardised mean difference.

### Creatine kinase MB (CK-MB) is a risk factor for ES following PCI in AMI

Three studies included in this research investigated the impact of CK-MB on ES following PCI in AMI. These studies encompassed a total of 402 patients, with 43 cases experiencing ES and 359 cases not experiencing it. Heterogeneity testing showed no significant variation among the studies (*P ═* 0.98, *I^2^* ═ 0% < 50%); thus, a fixed-effect model was applied for analysis. A notable increase in CK-MB levels was observed in patients with ES compared to those without (SMD ═ 0.91, 95%CI: 0.59–1.24, *P <*0.01), establishing CK-MB as a risk factor for ES following PCI in AMI (Figure 10A). The symmetry of the funnel plot indicated minimal publication bias (Figure 10B). The sensitivity analysis results, as shown in Figure 10C, revealed no significant change in study heterogeneity, indicating the meta-analysis outcomes regarding CK-MB are robust and reliable.

### Bradycardia is correlated with ES following PCI in AMI

**Figure 11.**
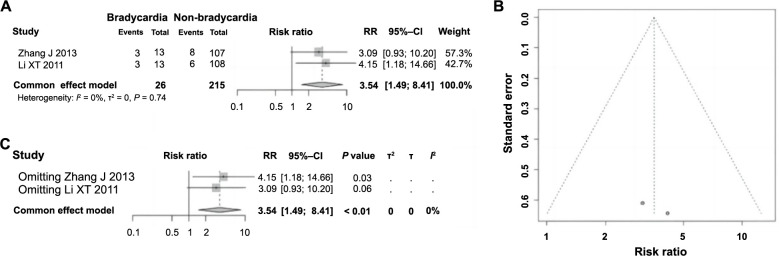
**Forest plot of meta-analysis on the association between bradycardia and ES following PCI in AMI.** (A) Forest plot of bradycardia and ES following PCI in AMI; (B) Funnel plot evaluating publication bias; (C) Sensitivity analysis. ES: Electric storm; PCI: Percutaneous coronary intervention; AMI: Acute myocardial infarction; RR: Relative risk; CI: Confidence interval.

Among the 11 included articles in this analysis, only two examined the impact of bradycardia on ES following PCI in AMI. Heterogeneity testing revealed no significant differences across the studies (*P* ═ 0.74, *I^2^* ═ 0% < 50%), warranting the use of a fixed-effect model for combined analysis. The probability of experiencing ES following PCI in AMI was significantly higher in patients with bradycardia compared to those without (RR ═ 3.64, 95% CI: 1.49–8.41, *P* < 0.01), demonstrating a clear correlation between bradycardia and the occurrence of ES, identifying it as a risk factor (Figure 11A). The funnel plot was essentially symmetrical, indicating no evident publication bias (Figure 11B). Further sensitivity analysis showed consistent homogeneity among the studies (Figure 11C), affirming the stability and dependability of the meta-analysis findings.

## Discussion

VT and ES represent malignant arrhythmic disorders due to instability in ventricular electrical activity. Causes of ES encompass a range of factors, including cardiomyopathies, acute myocardial ischemia, pharmacological agents, surgical interventions, channelopathies, and electrolyte imbalances. Among these, AMI has been identified as a principal trigger [19], concurrently serving as a significant precursor to sudden cardiac death [31]. The mechanism underlying this association involves the formation of a voltage gradient between ischemic and non-ischemic myocardial cells post-AMI, leading to abnormalities in cell membrane function, decreased membrane potential, shortened action potential duration, increased ectopic automaticity, and reduced refractory periods, thereby precipitating rapid arrhythmic events. Furthermore, reductions in action potential amplitude and Vmax, dispersion of refractoriness, and diminished conductivity contribute to reentrant ventricular arrhythmias [32]. Patients in the acute phase of myocardial infarction experience a peak period for ES onset, marked by myocardial hypoxia, injury, necrosis, and neuroendocrine alterations. Clinical observations have linked ES with elevated risks of all-cause mortality, cardiac transplantation, and acute heart failure hospitalizations [33]. PCI is a widely utilized and effective treatment for AMI; however, the clinical risk factors for ES following PCI in AMI patients remain unclear. Through a meta-analysis of domestic and international literature, this study has analyzed the risk factors for ES following PCI in AMI, offering robust evidence for predicting and managing clinical risks in such patients. The study facilitates the early prediction of ES risk, enabling the development of effective treatment strategies, early intervention, and precision medicine, thereby reducing the incidence and mortality rates of ES.

This meta-analysis identified ten risk factors for ES following PCI in AMI patients, including TIMI flow grades, cTnI, CK-MB, reperfusion arrhythmias, persistent hypotension, RCA as the IRA, IRA diameter, β-blocker usage, renal dysfunction, and bradycardia. TIMI flow grading serves as a benchmark for evaluating coronary reperfusion, with grade 0 indicating no perfusion due to occlusion, grade 1 suggesting slow flow without perfusion, grade 2 indicating partial perfusion, and grade 3 representing complete perfusion. Clinically, TIMI grades 0–1 are associated with myocardial ischemia or lack of coronary flow, while grades 2–3 suggest reestablished coronary flow. The clinical value of TIMI flow grading in assessing perfusion of the IRA has achieved consensus, with numerous studies reporting poor post-PCI coronary reperfusion as an independent risk factor for adverse outcomes in AMI patients [34, 35]. Research has demonstrated that TIMI flow grading significantly impacts in-hospital and long-term outcomes, such as mortality rates, with notably increased mortality observed in AMI patients with TIMI grades 0–1 post-PCI [36, 37].

Our findings indicate a significant correlation between TIMI flow grading and the incidence of ES. As TIMI flow grading increases, indicating improved reperfusion levels in ischemic myocardium, the risk of ES decreases. Absence or slow reflow in the “culprit” vessel suggests suboptimal blood flow perfusion post-coronary opening. Post-PCI TIMI grades 0–1 indicate coronary myocardial ischemia, exacerbating myocardial cell electrophysiological heterogeneity and elevating the risk of ventricular arrhythmias.

Suboptimal reperfusion leads to local microcirculation dysfunction, hindering collateral circulation development, damaging endothelial cells, disrupting vasodilator release, causing distal myocardial supply issues, exacerbating ventricular remodeling, and increasing ES risk [38, 39]. This risk is particularly high in patients with extensive preoperative coronary occlusions, who often experience severe ventricular remodeling and are more likely to have poor reperfusion post-PCI, thereby elevating the risk of ES complications [40]. Consequently, clinical practice should aim for the early, comprehensive, and sustained opening of the infarcted vessel to minimize the risk of concurrent ES.

Of note, cTnI forms an essential component of the cardiac troponin complex, existing in both complex and free forms within myocardial cells. Upon myocardial injury, cTnI is released into peripheral blood [41], displaying high sensitivity and specificity, with significant elevations detectable within one hour of ischemic damage, thereby serving as an effective early diagnostic marker for AMI [42]. Prior research on 232 older patients with acute heart failure found that cTnI levels were significantly higher in the critically ill group compared to the non-critically ill group, indicating that cTnI is an independent risk factor affecting prognosis [43]. CK-MB is a type of CK that exists in the myocardium of the human body. It is released into the peripheral blood during myocardial injury and has a high sensitivity and specificity. Previous studies have found that CK-MB levels will significantly increase within the first 6 h of myocardial infarction and reach their peak within 24 h. The content of CK-MB is positively correlated with the extent of myocardial ischemia [44].

This study, through meta-analysis, identified significant correlations between CK-MB and cTnI levels and the incidence of ES post-PCI in patients with AMI. It was found that higher levels of CK-MB and cTnI were associated with an increased risk of ES in these patients. CK-MB and cTnI are regarded as crucial clinical markers for assessing myocardial damage, with their elevated levels indicating more severe myocardial infarction or larger infarct size. However, research focusing specifically on the relationship between these biomarkers and ES is limited, with existing studies primarily concentrating on malignant arrhythmias and VT. The consensus on the relationship between CK-MB, cTnI, and malignant arrhythmias is not unified, but the majority view them as risk factors for VT or malignant arrhythmias. A previous study on 80 patients with chronic heart failure revealed a significant correlation between cTnI and malignant arrhythmias, suggesting cTnI as an effective predictor for malignant heart rate abnormalities in these patients [45]. Another study detected cTnI levels in 174 patients with chronic heart failure and monitored 24 h dynamic electrocardiograms, finding a close relationship between elevated cTnI levels, ventricular arrhythmias, the frequency of persistent VT episodes, and the prognosis of patients with chronic heart failure [46]. Furthermore, a prior investigation of 1120 consecutive hospitalized myocardial infarction patients indicated that those with persistent monomorphic VT had higher peaks of CK-MB enzyme activity, suggesting CK-MB as an independent predictor for persistent monomorphic VT and mortality [47]. Additional studies have found higher CK-MB activity to be a risk factor for persistent monomorphic VT [48–51]. However, further logistic regression analysis revealed they were not independent risk factors. ESs are triggered by instability in cardiac electrical activity, characterized by persistent VT or VF, classifying them under malignant ventricular arrhythmias. It is theorized that post-myocardial infarction, the release of cTnI and CK-MB into peripheral blood can stimulate the vagus nerve, leading to decreased heart rate and cardiac output, exacerbating myocardial ischemia and hypoxia. This condition further elevates cTnI and CK-MB levels, causing significant metabolic disparities between ischemic and normal myocardial regions. Such disparities induce inconsistencies in myocardial repolarization, increase the dispersion of ventricular refractory periods, and facilitate reentrant excitation, triggering multiple episodes of ventricular arrhythmias and culminating in ESs [52].

This study, utilizing meta-analysis, elucidated a significant correlation between reperfusion arrhythmias and the onset of ES in patients undergoing PCI for AMI. Among 340 patients experiencing reperfusion arrhythmias, 110 developed ES (32.35%), contrasting with only 32 occurrences of ES (8.27%) in 387 patients without reperfusion arrhythmias. This finding underscores reperfusion arrhythmias as a salient risk factor for ES following PCI in AMI. Prior research findings [52] suggest that the incidence of arrhythmias during the myocardial ischemia-reperfusion process can reach up to 80%, predominantly manifesting as VT and VF. Ischemic conditions lead to the closure of gap junctions between ischemic and normally perfused myocardium, slowing conduction and increasing heterogeneity, which, combined with asynchronous dispersion of action potential repolarization, can provoke unidirectional conduction block and reentrant excitations, culminating in ES. The release of large quantities of renin from hypertrophied myocytes activates the renin–angiotensin system, leading to an influx of calcium ions into myocardial cells through L-type calcium channels, causing intracellular calcium overload and increasing the risk of arrhythmias [53]. Elevated catecholamine levels following AMI exacerbate sympathovagal imbalance, further elevating the risk of arrhythmias. The washout and release of lactate accumulated in myocardial cell gaps during reperfusion after PCI cause a shortening of the refractory period in reperfused myocardial tissue. Concurrently, the resurgence of blood flow introduces oxygen, free radicals, and superoxide radicals, initiating a cascade that alters ion pump activity on the cell membrane, disrupting intracellular and extracellular ion distribution and local electrophysiology, destabilizing myocardial cell potential, and lowering the threshold for VF, leading to persistent ventricular arrhythmias and ultimately ES [54].

A meta-analysis found that out of 605 patients with persistent hypotension, 132 cases (21.82%) experienced an ES. Of the 5863 patients who did not experience persistent hypotension, 274 cases (4.67%) developed ESs. Therefore, it can be concluded that there is a significant correlation between persistent hypotension and ESs, which is a risk factor for the occurrence of ESs in patients with AMI after PCI. This is because the myocardial electrical activity of patients with myocardial infarction is unstable. Hypotension, especially persistent hypotension, can lead to VF and trigger an ES.

Moreover, the meta-analysis revealed a marked correlation between persistent hypotension and the occurrence of ES, identifying it as a significant risk factor for ES following PCI in AMI. Among 605 patients with persistent hypotension, 132 (21.82%) experienced ES, compared to 274 (4.67%) out of 5863 patients without persistent hypotension. This significant correlation is attributed to the instability of myocardial electrical activity in myocardial infarction patients, where particularly persistent hypotension can precipitate ventricular fibrillary vibrations, triggering ES. Additionally, the analysis highlighted a notable association between the RCA as the IRA and the incidence of ES, deeming it a risk factor for ES following PCI. Among 124 patients with the RCA as the IRA, 28 (22.58%) developed ES, compared to 15 (5.40%) out of 278 patients with other arteries as the infarct-related site. Previous data have shown that patients with RCA occlusion exhibit a higher proportion of reperfusion arrhythmias during PCI and chronic arrhythmias compared to those with occlusions in other coronary branches [55, 56]. The significance of RCA as the primary blood supply to the right atrium and ventricle means that acute occlusion can diminish blood perfusion to the sinoatrial and atrioventricular nodes, impairing pacing functions and conduction, leading to bradycardia or even asystole prolonged ventricular asystole extends the Q-T interval, increases sodium influx, and reduces potassium efflux, elongating ventricular repolarization, destabilizing cardiac electrophysiology, and facilitating reentrant rapid arrhythmias that precipitate ES [57].

This study, through meta-analysis, discovered a significant correlation between the diameter of the IRA and the occurrence of ES following PCI in patients with AMI. As the diameter of the “culprit” vessel increases, so does the risk of ES, likely due to the influence of the vessel’s diameter on the extent of myocardial ischemia. Typically, a larger vessel diameter means more myocardial cells are supplied and affected. When infarction occurs in such a vessel, a larger number of myocardial cells are compromised, leading to more severe myocardial involvement. Greater myocardial damage increases the probability of electrical instability during the ischemia-reperfusion process, leading to the potential onset of ES.

The meta-analysis further revealed that among 7191 patients administered β-blockers, 409 experienced ES (incidence rate of 5.69%), whereas among 897 patients not administered β-blockers, 103 experienced ES (incidence rate of 11.48%). This outcome indicates that β-blocker usage can significantly reduce the incidence of ES, serving a protective role. A meta-analysis highlighted that β-blockers could lower the incidence of VF in heart failure patients [58]. Numerous clinical studies have reported that β-blocker usage can improve patient outcomes, reducing the incidence of complications and mortality. β-blockers reduce sympathetic nervous tension and catecholamine levels, counteracting the toxicity of catecholaminergic neurotransmitters and enhancing cardiac vagal tone, thereby lowering the risk of ES.

In this study, among 427 patients with renal dysfunction, 81 experienced ES (18.97%), whereas among 7661 patients without renal dysfunction, 431 experienced ES (5.63%). Thus, a significant correlation exists between renal dysfunction and ES, marking it as a risk factor for ES following PCI in AMI. Research on the relationship between renal function and ES is limited, yet numerous reports indicate that renal dysfunction is a risk factor for poor outcomes in STEMI patients [59]. Specifically, male AMI patients with renal dysfunction face a 3.771 times higher risk of all-cause mortality compared to those without renal dysfunction, with a 2.2992 times higher risk of major adverse cardiovascular events [60]. Renal dysfunction may lead to fluid retention, increasing cardiac load and impacting cardiac contractility. Studies show a close association between renal dysfunction in AMI patients and lower ejection fractions, with patients having lower ejection fractions experiencing significantly higher mortality rates. Moreover, research tracking 1400 consecutive cases of patients with VF found that an estimated glomerular filtration rate (eGFR) < 82.5 mL/min/1.73m^2^ was a good predictor of VF recurrence [60]. This condition may be ascribed to the higher levels of creatinine and urea in their bodies, the accumulation of which exacerbates acidosis and disturbs calcium-phosphate metabolism, interfering with Na^+^/K^+^ balance in patients with renal dysfunction, which increases the risk of myocardial damage and electrical instability, and thus induce ES and arrhythmias.

Furthermore, among 26 patients with bradycardia, six experienced ES (23.08%), while among 215 patients without bradycardia, 14 experienced ES (6.51%). This significant correlation indicates that bradycardia is a risk factor for ES following PCI in AMI. Bradycardia is a common complication of AMI, leading to sinus bradycardia, sinus arrest, sinoatrial block, and atrioventricular block, all of which can induce bradycardia and serve as a pathological basis for ES. This finding elucidates why bradycardia emerges as a risk factor for ES following AMI [61].

It is noteworthy that research has demonstrated the efficacy of AI in managing diseases related to coronary artery and atrial fibrillation, facilitating ease in patient risk assessment, diagnosis, treatment selection, procedural guidance, and remote monitoring [62]. Therefore, utilizing AI algorithms to identify risk factors for ES following PCI in patients with AMI could enhance precision medicine in AMI management, thereby improving treatment outcomes and reducing mortality. Despite the promising application prospects of AI, its development is constrained by the need for extensive, high-quality data.

**Figure 12.**
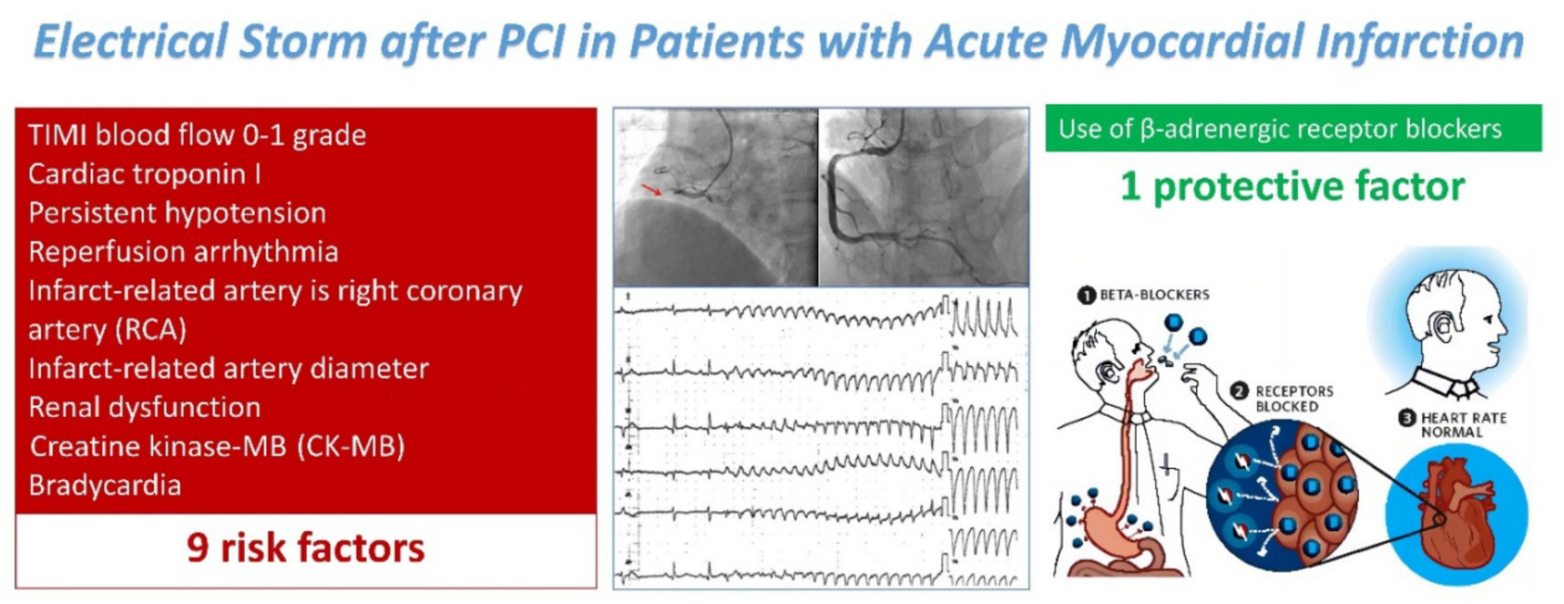
**Risk and protective factors for ES following PCI in AMI.** ES: Electric storm; PCI: Percutaneous coronary intervention; AMI: Acute myocardial infarction; RCA: Right coronary artery; CK-MB: Creatine kinase-MB.

However, due to the limited literature available on risk factors for ES following PCI in AMI, this study’s meta-analysis incorporated a restricted number of publications and case counts. Furthermore, the inclusion of primarily Chinese-language publications, with fewer English-language studies, may introduce regional and publication biases, potentially affecting the quality of evidence in the meta-analysis. Additionally, this study exclusively included cohort studies, lacking cross-sectional research. For certain risk factors, heterogeneity was detected among the included studies. Thus, a random effects model was employed to derive relatively conservative conclusions. Beyond sensitivity analysis, this study did not conduct further subgroup analyses to explore the sources of heterogeneity. Moreover, due to the singular mention in the literature, a meta-analysis on some risk factors could not be conducted, leaving their status as risk factors for ES following PCI in AMI unresolved. Consequently, there is a need for more in-depth, global research to expand the study scale and increase sample diversity, thereby supporting our conclusions and enhancing the study’s credibility and accuracy for clinical application.

## Conclusion

In summary, TIMI flow grades of 0–1, levels of cTnI, persistent hypotension, reperfusion arrhythmias, the RCA as the IRA, the diameter of the IRA, renal dysfunction, levels of CK-MB, and bradycardia are identified as risk factors for the occurrence of ES following PCI in patients with AMI. Patients presenting these risk factors warrant special clinical attention. Early risk assessment for these factors is crucial for reducing mortality rates in AMI patients. Furthermore, the use of β-blockers is recognized as a protective factor against ES following PCI in AMI (Figure 12).

## Data Availability

Data underlying this article will be shared on reasonable request to the corresponding author.
